# The Transplantation of Bone

**Published:** 1918

**Authors:** 


*The Transplantation of Bone*. By W. E. Gallie and D. E. Robertson. Abs. from the *Jour. A. M. A.* April 20, 1918.

The writers declare that bone transplantation in clinical practice has been put on a sound scientific basis. They report results from four years of experimentation and clinical experience.

They conclude that although an autogenous bone-graft may result in the death of all the cells in the lacunae and most of those in the Haversian canals, nevertheless on the surface of the graft and in the open mouths of the Haversian canals, many osteoblasts persist and absorb nutriment from the bathing lymph, and therefore proliferate and produce those changes that make autogenous grafts valuable in surgery. Thus new bone is laid down and old bone replaced. These changes and the replacement are slow in hard dense bone such as the crest of the tibia, but very rapid in open cancellous bone, such as a rib.

Of great importance is the abundance of osteoblasts on the surface of the graft. If the endosteal and periosteal surfaces are undisturbed and exposed to a good supply of lymph, the changes occur with certainty and rapidity. If endosteal and periosteal surfaces are removed, very little osteogenesis takes place from the graft itself because of the scarcity of the osteoblasts.

To insure success in the autogenous grafts, it is necessary to supply living osteoblasts in the largest possible quantity. Living osteoblasts survive only on the surfaces. It the tibia is used for cutting the graft it should be taken, not from the crest, but from the face of the bone, and the width of the graft should be made greater than its thickness. The old theory, that grafts should be cut from the solid crest of the tibia, has been wholly refuted by the authors' experiments. It is much better to avoid the tibia, when strength is not the primary requisite, and to use a rib slotted so as to expose the endosteal surface to the supply of the lymph. The amount of bone regeneration from a rib is much greater than that from the long bones. It is wiser to split the graft longitudinally into a number of pieces, since in this way more bone surface will be exposed and more osteoblasts will survive.

In operating for an ununited fracture in a long bone, the authors insert a single graft strong enough to fix the fragments and then fill in around it with a number of smaller strips. They believe the periosteum is not essential to successful transplanting.

When the autogenous graft is not essential and when fixation is the chief object of the operation, the authors recommend the boiled bone graft as having decided advantages. At the end of two months the union of the dead to the living bone is solid. The changes that take place in the autogenous graft also take place in the boiled bone graft. New bone is laid down on the surface of the graft and the dead bone is gradually replaced by the living.

These observations were first made in experimentation but similar results were obtained later in clinical practice. In 60 cases of Pott's disease a spinal graft operation was performed. Also in cases of non-union or mal-union autogenous grafts were used. In 60 or more recent fractures boiled bone plates and screws, or screws alone, were used.

In the open operation for non-union, the writers favor the inlay graft as described by Albee, preferring it to the medullary plug, which obstructs the canal and prevents the outflow of osteoblasts. They drill holes and make sawcuts in the ends of the fragments to stimulate the inflammatory reaction which is necessary to osteogenesis.

